# Using standardized patients to assess the quality of type 2 diabetes care among primary care providers and the health system: Evidence from rural areas of western China

**DOI:** 10.3389/fpubh.2022.1081239

**Published:** 2022-12-22

**Authors:** Yuju Wu, Ruixue Ye, Chang Sun, Sha Meng, Zhengjie Cai, Linhua Li, Sean Sylvia, Huan Zhou, Lucy Pappas, Scott Rozelle

**Affiliations:** ^1^West China School of Public Health and West China Fourth Hospital, Sichuan University, Chengdu, Sichuan, China; ^2^Department of Operation Management, West China Hospital, Sichuan University, Chengdu, Sichuan, China; ^3^Department of Health Policy and Management, Gillings School of Global Public Health, University of North Carolina at Chapel Hill, Chapel Hill, NC, United States; ^4^Freeman Spogli Institute for International Studies, Stanford University, Stanford, CA, United States

**Keywords:** quality of type 2 diabetes care, standardized patients, healthcare system, rural China, primary care

## Abstract

**Background:**

Improving type 2 diabetes (T2D) care is key to managing and reducing disease burden due to the growing prevalence of diabetes worldwide, but research on this topic, specifically from rural areas, is limited. This study uses standardized patients (SPs) to assess T2D care quality among primary care providers to access the healthcare system in rural China.

**Methods:**

Using multi-stage random sampling, health facilities, providers, and households were selected. SPs were used to evaluate providers' T2D care quality and a questionnaire survey was used to collect patient sorting behaviors from households. Logistic regression was used to explore factors correlated with T2D care quality. Provider referral and treatment rates were combined with patient sorting behaviors to assess the overall quality of T2D management by rural China's healthcare system.

**Results:**

A total of 126 providers, 106 facilities, and 750 households were enrolled into this study. During SP interactions, 20% of rural providers followed the national guidelines for T2D consultation, 32.5% gave correct treatment, and 54.7% provided lifestyle suggestions. Multi-variable regression results showed that providers who had earned practicing certificates (β = 1.56, 95% CI: 0.44, 2.69) and saw more patients (β = 0.77, 95%: 0.25, 1.28) were more likely to use a higher number of recommended questions and perform better examinations, whereas providers who participated in online training were less likely to practice these behaviors (β = −1.03, 95%: −1.95, −0.11). The number of recommended questions and examination (NRQE) was the only significant correlated factor with correct treatment (marginal effect = 0.05, 95%: 0.01, 0.08). Throughout the rural healthcare system, 23.7% of T2D patients were treated correctly.

**Conclusion:**

The quality of T2D care in rural western China, especially throughout the consultation and treatment process during a patient's first visit, is poor. Online training may not improve T2D care quality and low patient volume was likely to indicate poor care quality. Further research is needed to explore interventions for improving T2D care quality in rural China's healthcare system.

## Introduction

Diabetes is a leading cause of mortality and reduced life expectancy around the world. From 1990 to 2017, the global prevalence of diabetes increased more than 129% from 211 to 476 million, and the number of global deaths due to diabetes increased more than 125% from 0.61 million deaths to 1.37 million (1). Type 2 diabetes (T2D), the most common type of diabetes, accounts for ~90% of all diabetes cases internationally (2). In response to the major disease burden diabetes presents, organizations worldwide have set forth measures for decreasing diabetes' prevalence. For example, the United Nations Sustainable Development Goals (SDG) included the aim to reduce premature mortality from non-communicable diseases (NCDs), including diabetes, by one-third by 2030 (3). Similarly, the World Health Organization's (WHO) Global Action Plan for the Prevention and Control of NCDs has proposed a series of measures for the surveillance, prevention, and control of diabetes and its complications, as well as specific measures for diagnosing and treating T2D (4).

In 2019, China reported having the largest number of adults with diabetes in the world (116.4 million) and predicted that this number would increase to 147.2 million by 2045 (5). In order to decrease the prevalence and reduce the complications associated with diabetes, China implemented a comprehensive healthcare policy and has established 265 national demonstration areas where short-term pilot programs aim to promote better healthcare practices as well as better detection methods for controlling the prevalence of chronic diseases (6). Additionally, China has further developed healthcare system integration throughout the country to promote the flow of healthcare resources to primary care facilities for long term health programming (7).

Healthcare systems play a foundational role in dealing with the increasingly high prevalence of non-communicable diseases (8), including diabetes. In China, the rural healthcare system is comprised of three tiers of healthcare providers: village clinics (VC), township health centers (THC), and county hospitals (CH). Under China's guidelines for diabetes management within the rural healthcare system, primary care providers from VCs and THCs take primary responsibility in diagnosing patients during their initial visits and treating the diagnosed patients. First-time patients are recommended to visit a VC or THC, and to then visit a CH if their health issues remain unresolved (9). As a result of these healthcare system developments, diabetes prevention and treatment services in China have been gradually transferred from city hospitals to local primary care providers, which has improved diabetes patients' access to diagnosis and treatment (10).

Despite patients' improved accessibility to diabetes healthcare services, the complex phenotypes and multiple needs of individuals with diabetes requires high quality diabetes care and a healthcare system designed to reduce the burden of this NCD (11). However, previous studies on the quality of diabetes care (that analyzed the counseling and examination process quality, the treatment quality, and subsequent health outcomes) have identified suboptimal performance during initial case diagnosis, evidenced by, for example, a lack of essential examinations (12, 13). Furthermore, for T2D in particular, there is little known about the differences in healthcare system quality in regards to a patient's choice of healthcare facility for their first visit (i.e., whether a patient chooses to first visit a VC or a THC). This is particularly relevant given the gaps in T2D care quality between the different tiers of providers. Combined with the increasing burden on primary care providers for managing T2D, understanding the quality of T2D care and how it may vary by a patient's visiting behavior among China's three-tier rural healthcare system is vital to identifying areas for improvement.

One of the fundamental problems with assessing the quality of T2D care is the methodological limitations of previous studies; for example, studies that had used electronic medical records or questionnaire survey data on patient care outcomes often found the method to lead to incomplete and inaccurate results (14). Instead, the standardized patients (SPs) method has become considered as the “gold standard” for evaluating the quality of clinical practice (15). SPs are individuals recruited from local communities and trained to present consistent disease cases to healthcare providers. There are three distinct advantages to assessing clinical practice with SPs compared to other commonly used methods (15, 16). First, because providers are unaware they are being assessed during SP interactions, clinician behavior remains unbiased, compared to the direct observation method which has been found to introduce Hawthorne effects to a provider's observed behaviors (16, 17). SPs can also reduce or eliminate recall bias compared to the method of surveying actual patients after a visit. Second, SPs can measure “real” clinical practice as opposed to only measuring clinical knowledge (which is commonly examined using clinical vignettes or a questionnaire survey, leading to providers being aware of being assessed). Third, because the SP approach examines complete interactions between providers and mock patients, the referral process between the different tiers of healthcare facilities may be observed in practice; these observations may then be used to evaluate the quality of the overall healthcare system. The SP approach has been used in China and internationally to investigate how diseases such as diarrhea, unstable angina, and tuberculosis are treated (18–20). However, to our knowledge, few studies have used SPs to evaluate the quality of T2D care, especially in rural China where the prevalence of diabetes increases by 2.5% points annually (21).

In order to fill this identified gap in the literature, the present study has three objectives. First, we use SPs to assess the quality of T2D care among primary healthcare providers in rural China. Second, we explore the correlates of the quality of T2D care and seek to provide policy or healthcare reform implications for improving the quality of T2D care in rural China. Third, we combine patients' facility sorting behaviors and provider-level quality analyses of T2D to determine the ability of China's rural healthcare system for effectively managing diabetes.

## Materials and methods

### Setting and sampling

**Figure 1:**
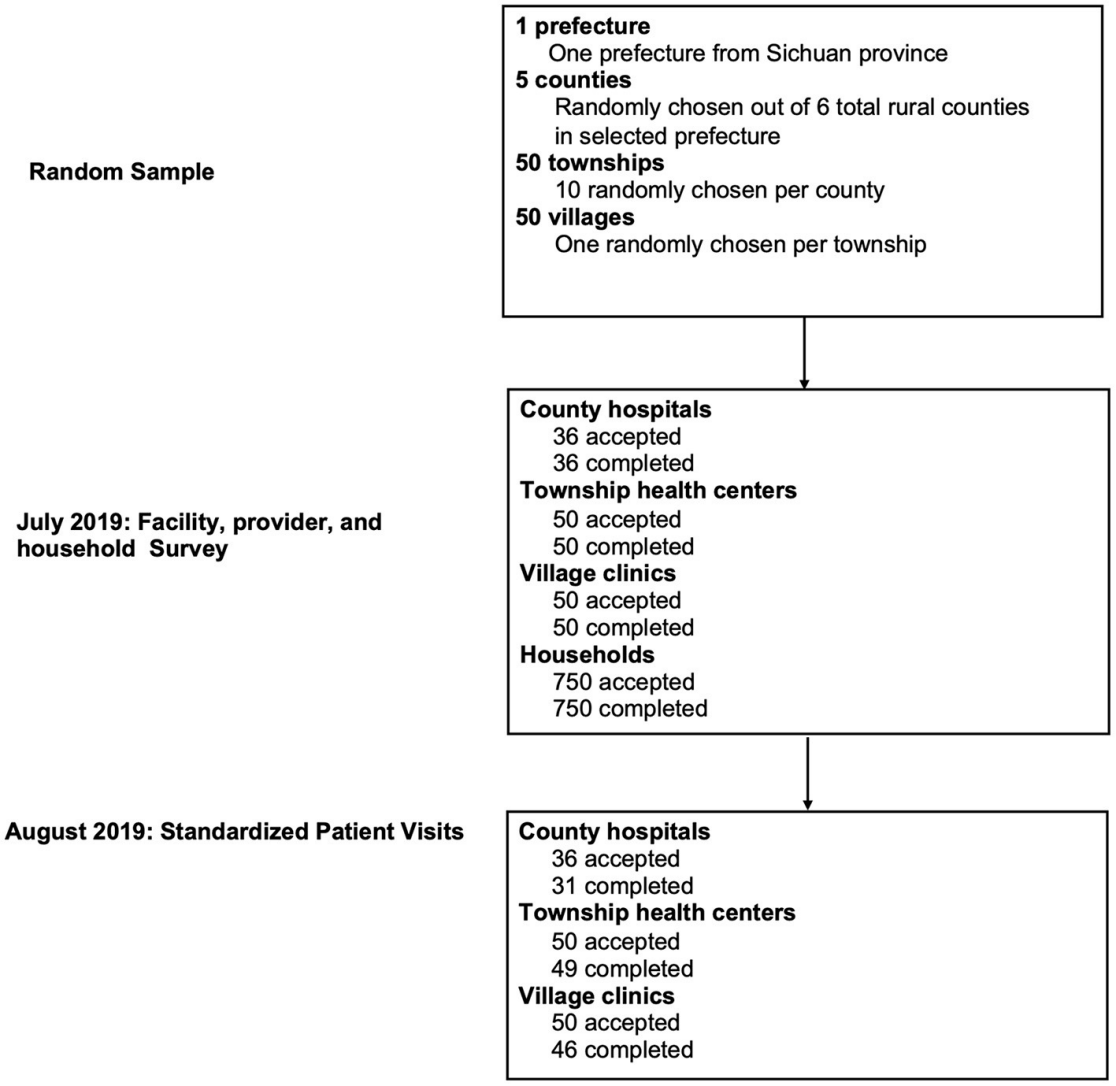
STROBE flowchart. Standardized patients were randomly assigned to facilities and within each facility, standardized patients visited the provider following normal procedures for walk-in patients. Fifteen households were randomly selected from the resident roster of each sample village, totaling 750 households.

The facilities and healthcare providers sampled for this study were selected from rural areas in one prefecture of Sichuan Province in western China. The sample was chosen using a four-stage random sampling procedure. First, five counties were randomly selected from the prefecture. Second, 50 townships were randomly selected from these five sample counties. Third, the sample was then evenly divided with 10 townships chosen from each county. Fourth, one village was randomly selected from each sample township. In total, 50 villages from the sample townships were enrolled into the study. All providers of general and internal medicine who were on duty on our survey day from the sample facilities within the sample villages were surveyed. From this sampling process, a total of 126 providers were included in the study (see Figure 1).

### Data collection

#### Provider survey

Structured questionnaires were administered to collect provider information. Trained investigators interviewed providers to obtain information regarding their age, gender, medical education, qualifications, medical experience, income, and undergraduate major.

#### Household survey

Residential-level demographics—including household socio-demographic characteristics, whether a member of the household had been diagnosed with diabetes, and the facility-sorting behavior of the household members (i.e., whether they choose to first visit a VC or a THC)—were collected by trained investigators through face-to-face interviews. The choice of which facility a patient visits first is important in evaluating the quality of China's rural healthcare system in diagnosing and treating T2D, as this choice determines how the patient will be diagnosed and initially treated, which subsequent facilities they will visit through referrals, and the efficacy of the treatment process as a whole. Therefore, all households were asked which facility a member would choose to visit first when they experienced symptoms related to diabetes, such as eating more, drinking more, and unexplained weight loss.

### Standardized patients

Provider quality was assessed through their interactions with incognito SPs. T2D is a disease that qualifies for being assessed by the SP methodology as (1) there are no obvious physiological symptoms of T2D and (2) there is low risk that SPs will be exposed to invasive procedures or tests during an initial examination. Thus, using SP interactions, we assessed providers' process quality and the accuracy of their diagnosis and treatment. The standards of quality assessment we used were taken from Chinese clinical guidelines (22), which are presented in Appendix Text 1.

SPs were recruited from local communities and selected through interviews with our research team to confirm that their demographic characteristics matched the standardized T2D case profile. All selected SPs were trained in a classroom setting for 1 week by a team of researchers and consulting medical professionals in order to present consistent T2D cases. Classroom training focused on preparing SPs to represent the T2D case to providers in a consistent and unsuspicious manner. Medical professionals discussed the symptoms of T2D to be portrayed by SPs and the typical behavior and presentation of real patients afflicted with the diseases. Following classroom instruction, the SPs went through extensive field rehearsals in rural areas with clinicians who volunteered to assist with the study.

All SPs followed a standardized case script which was developed by our research team in conjunction with diabetes specialists. To adapt the case script to the local context, local diabetes prevention and management authorities were also included in the consultations. The details of the case script can be found in Appendix Text 2. To prevent invasive testing on an SP, such as a Fasting Plasma Glucose test, every SP carried a prepared blood glucose test report form with them for their visit. If a provider asked them to take an invasive examination, the SP would present the test report to them.

#### SP visits

SPs visited the target providers in late August 2019. One SP visited one provider in each sample facility. Each SP was randomly assigned to a provider to reduce the potential for any differences in individual SP presentations and to reduce bias comparisons across providers. Upon entering each clinic, the SP was seen by any provider who was available at the time. In other words, SPs made no attempt to be seen by specific providers.

We used three methods to collect data on SP-provider interactions. First, SPs wore a concealed recording device. This allowed the research team to accurately evaluate interactions without relying on the SP's ability to recall details. Second, SPs were administered a case-specific “debriefing survey” upon exiting clinics. This survey covered the SP's interaction with the provider, the SP's own impressions of the provider, and any additional observations made by the SP that they thought were relevant but not captured on the audio recording. Finally, to collect information on any drugs dispensed, SPs were directed to purchase all medications prescribed to them by the provider.

### Statistical analysis

We calculated the means or proportions across all interactions for each of our primary outcomes: (1) the SP-provider consultation process as determined by the number of recommended questions and examinations (henceforth referred to as NRQE) asked and conducted by the provider; (2) the correct diagnosis as determined by the accuracy and completeness of the provider's diagnosis; and (3) the correct treatment as determined by the overall accuracy of the provider's prescribed treatment. All outcome variables were evaluated according to China's national clinical guidelines (22). To assess the correlates of these variables, we used ordinary least squares (OLS) regression for the NRQE variable (continuous variable) and logistic regression for the correct diagnosis and correct treatment variables (binary variables). For each outcome, we assessed correlations with a fixed set of facility-level and provider-level characteristics hypothesized to be related to T2D care quality.

We simulated system-level results by combining patient sorting behavior data with provider competence data from VCs and THCs to build a T2D management chain that represented the entire rural healthcare system. We used the patient sorting behavior data collected by the household survey to determine which facility a patient would visit first; we then used T2D treatment and referral data at the facility level to determine whether a patient would be treated correctly at that facility or to where the patient would be referred for further treatment. For instance, if a patient initially visited a VC, we used the correct treatment data and referral data at the VC tier (and, subsequently, the THC level) to calculate the probability of diabetes being correctly managed within the rural healthcare system. All analyses were conducted using STATA 14.0 (StataCorp, College Station, TX, USA).

## Results

### Provider characteristics

Table 1 shows the characteristics of all sampled providers. Out of the total 126 providers who were enrolled into our study, 36.5% (46/126) were from VCs, 24.6% (31/126) were from CHs, and the remainder were from THCs (38.8% or 49/126). VC providers had a lower education level than providers from THCs and CHs (*P* < 0.001). Moreover, VC providers had fewer practicing certificates; only 23.9% of VC providers held a practicing certificate, which was a significantly smaller proportion than providers working in THCs and CHs (91.8 and 96.8%, *P* < 0.001), respectively. During their undergraduate studies, 56.5% of VC majored in western medicine, compared to 79.6 and 77.4% of providers from THCs and CHs, respectively (*P* < 0.05). There were also significant income gaps between providers in the three tiers. The income of THC providers was twice the income of VC providers, and the income of CH providers was three times the income of VC providers (*P* < 0.001). Finally, 80.6% of CH providers had participated in online training, which was a significantly higher proportion than that of THC and VC providers (*P* = 0.005).

**Table 1:** Providers characteristics across three tiers of China's rural healthcare system.

**Provider characteristics**	**VC**	**THC**	**CH**	***P*-values**
Age (SD)	48.1 (7.9)	44.7 (8.4)	49.5 (11.8)	0.055
Gender (1 = male)	34 (73.9%)	32 (65.3%)	15 (48.4%)	0.071
Education (1 = bachelor's degree or higher)	0 (0.0%)	7 (14.3%)	11 (35.5%)	< 0.001
Practicing certificate (1 = yes)	11 (23.9%)	45 (91.8%)	30 (96.8%)	< 0.001
Major (1 = western medicine)	26 (56.5%)	39 (79.6%)	24 (77.4%)	0.030
Income (Yuan)	2,358.3 (995.9)	4,563.1 (1,352.9)	7,151.6 (3,277.1)	< 0.001
Diabetes patients in past 2 weeks (numbers)	5.2 (6.5)	6.3 (5.7)	26.6 (41.5)	< 0.001
Diabetes training 1 time per month or more (1 = yes)	11 (23.9%)	7 (14.3%)	6 (19.4%)	0.49
Online training (1 = yes)	20 (43.5%)	27 (55.1%)	25 (80.6%)	0.005
Sample size	46	49	31	

Source: Author's survey.

VC, Village Clinic; THC, Township Health Center; CH, County Hospital; SD, Standard deviation.

### Quality of SP-provider interaction process, diagnosis, and treatment

Results on the quality of T2D care among providers in the rural healthcare system are reported in Table 2. On average, providers used six of the 31 recommended questions and examinations (20%); no significant differences were found among the three tiers of providers (all *P*-values more than 0.05). The providers asked an average of 3.3 recommended questions out of the 19 questions required by the national clinical standards for diagnosing T2D, and performed 2.8 of the 13 required examinations. The correct diagnosis rate was more than 90% among providers from THCs and CHs, whereas only 69.6% of VC providers made correct diagnoses (*P* < 0.01). Overall, the correct treatment rate was generally below 40% and did not differ significantly among providers from the three tiers of facilities; however, when it came to prescribing the appropriate drugs, providers from VCs prescribed fewer correct drugs (41.4%) and more harmful drugs (23.9%) to patients than providers from both THCs (78.6 and 4.1%) and CHs (83.3 and 0%; all *P* < 0.05). Regarding non-pharmaceutical treatment plans, about 50% of all providers gave lifestyle suggestions to their patients, and no significant differences were found between providers in all three tiers (all *P*-values > 0.05). Regarding rates of referral, 37% of patients were referred from VCs to higher-tier facilities, whereas only 3.2% of patients were referred from CHs to other facilities (*P* < 0.05).

**Table 2:** Quality of type 2 diabetes care among providers from China's rural healthcare system.

**Diabetes care quality**	**VC**	**THC**	**CH**	***P*-values**
**Process quality**				
Number of recommended questions	3.0 (2.0)	3.5 (1.7)	3.3 (1.8)	0.43
Number of recommended examinations	2.5 (1.0)	2.9 (1.1)	2.9 (1.2)	0.13
Number of recommended questions and examinations (NRQE)	5.5 (2.5)	6.4 (2.2)	6.3 (2.3)	0.15
Average percent of recommended questions and examinations (ANRQE)	19.7% (6.2%)	20.2% (7.0%)	19.6% (7.1%)	0.91
**Diagnosis quality**				
Correct diagnosis	32 (69.6%)	44 (93.6%)	28 (93.3%)	0.002
**Treatment quality**				
Correct treatment	13 (28.3%)	18 (36.7%)	10 (32.3%)	0.68
Number of drugs dispensed (if any)	2.0 (1.1)	1.6 (1.2)	1.2 (0.4)	0.24
Correct drugs (if any)	12 (41.4%)	11 (78.6%)	5 (83.3%)	0.027
Harmful drugs (if any)	11 (23.9%)	2 (4.1%)	0 (0.0%)	< 0.001
Lifestyle guidance (if any)	24 (52.2%)	31 (63.3%)	14 (45.2%)	0.26
Monitoring blood glucose (if any)	19 (41.3%)	31 (63.3%)	20 (64.5%)	0.051
Referral	17 (37.0%)	7 (14.2.0%)	1 (3.2%)	0.017
Sample size	46	49	31	

Source: Author's survey.

VC, Village Clinic; THC, Township Health Center; CH, County Hospital.

**Figure 2:**
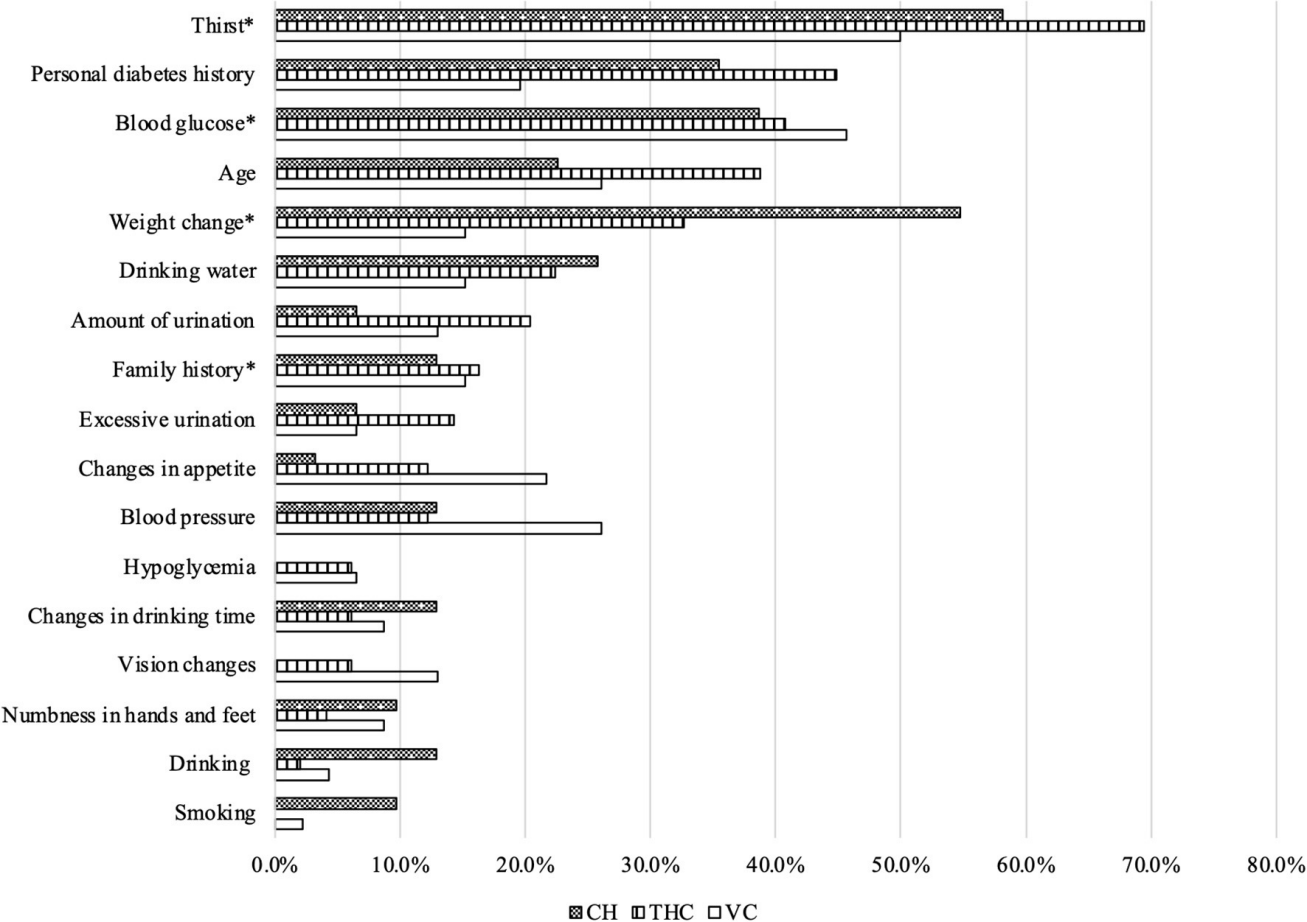
Recommended questions used during standardized patient-provider interactions. Source: Author's survey. CH, County Hospital; THC, Township Health Center; VC, Village Clinic; *Essential questions.

**Figure 3:**
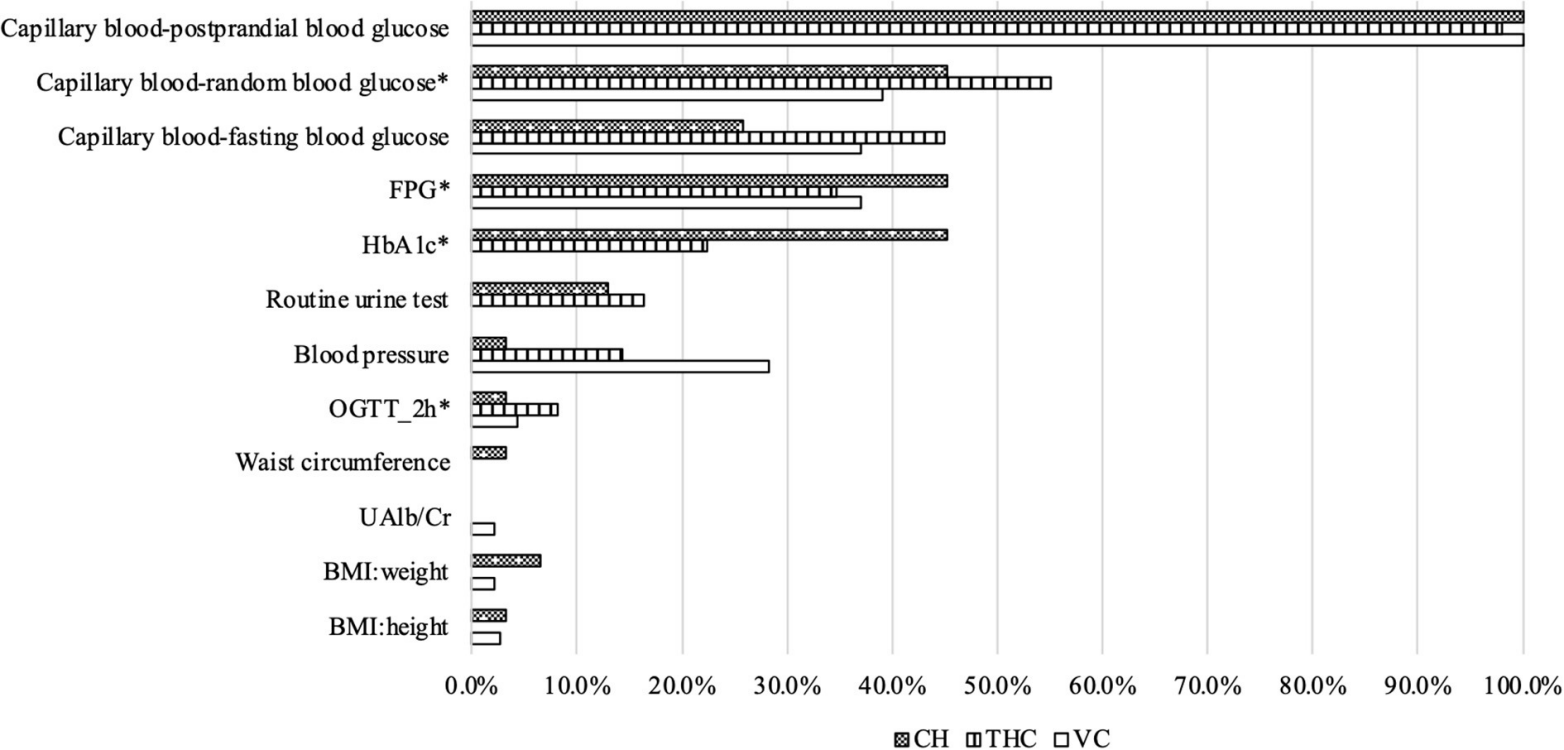
Recommended examinations used during standardized patient-provider interactions. Source: Author's survey. CH, County Hospital; THC, Township Health Center; VC, Village Clinic; *Essential examinations.

Turning to the frequency of diagnostic questions asked and examinations performed during SP-provider interactions (Figures 2, 3), more than half of the providers asked the recommended question of whether patients experienced “dry mouth and thirst.” Additional recommended questions were about the patient's history of diabetes, if they had previously received blood sugar testing, their age, and any weight changes they experienced in recent months; however, <50% of all providers asked these specific questions to the SPs. Moreover, <20% of all providers asked whether the patient had a family history of diabetes. Nearly all providers conducted capillary blood-postprandial blood glucose tests. Other essential exams were conducted infrequently. About 40% of all providers conducted FPG (Fasting Plasma Glucose) and HbA1c (hemoglobin A1c) tests. Few clinicians (around 5%) conducted the OGTT (Oral Glucose Tolerance Test), a golden standard for T2D diagnosis. Furthermore, although also considered essential for diagnosing T2D, no THC providers conducted the BMI exam, and very few providers (<5%) from VCs and CHs measured the weight and height of the SPs.

### Correlates of T2D care quality

**Figure 4:**
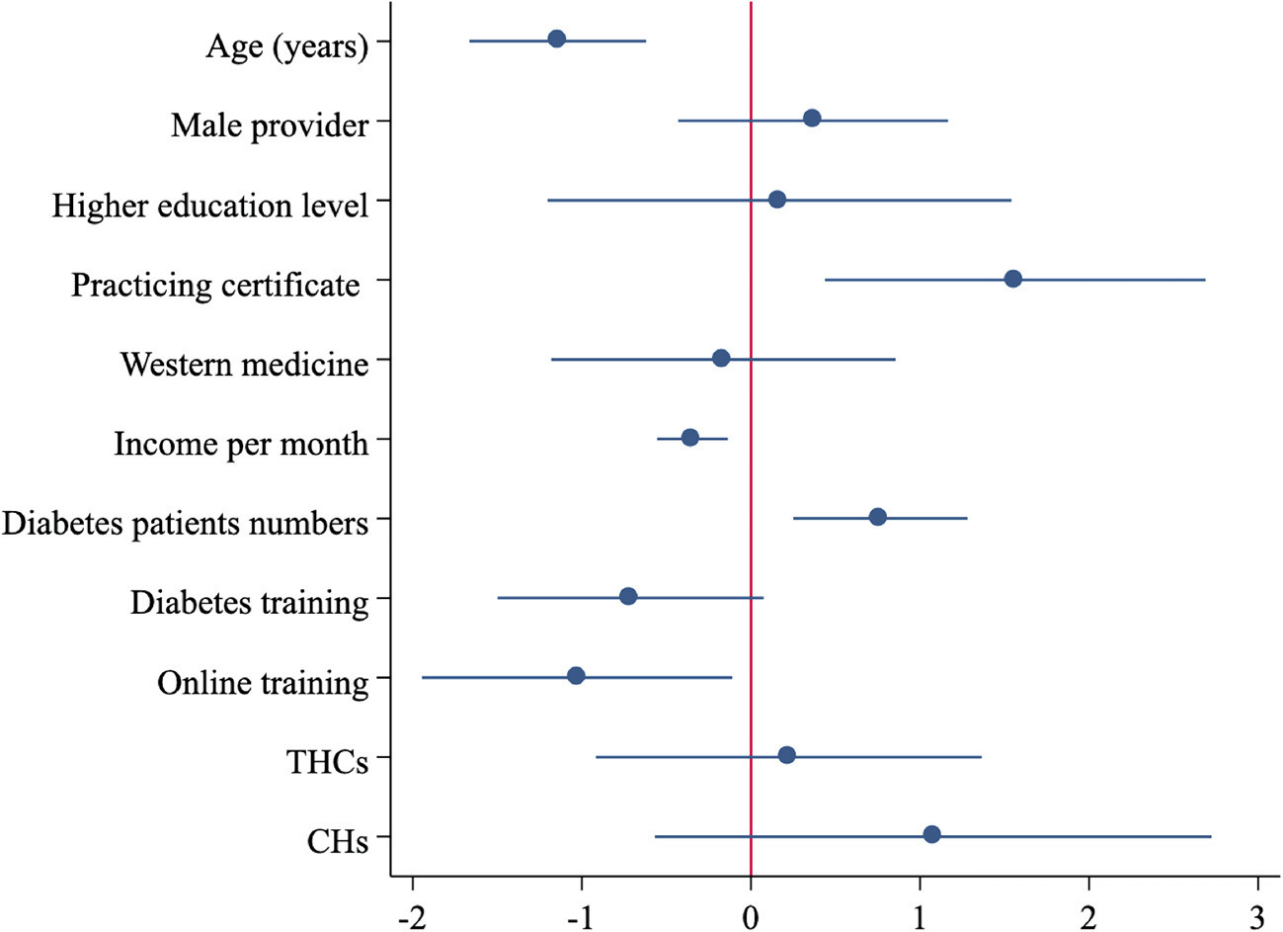
Associated factors for correct treatment of type 2 diabetes among rural clinicians. Source: Author's survey. NRQE, number of recommended questions and examinations; CH, County Hospital; THC, Township Health Center.

Figure 4 presents the results of the multivariate analysis assessing the correlates for provider process quality. Older providers used 1.14 fewer NRQE than younger providers, while providers with practicing certificates used 1.56 more NRQE than those without practicing certificates. Providers who had more patient visits in the past 2 weeks conducted 0.77 more NRQE. However, providers who had higher incomes addressed 0.35 fewer NRQE than those with lower incomes. Furthermore, providers who participated in online training in the past year conducted 1.03 fewer NRQE than those who did not participate in online training.

Appendix Figure 1 shows the correlates for correct diagnosis among providers. More NRQE addressed during SP-provider interactions was correlated with a higher probability of correct diagnosis by the provider. Similar to the correlates of process quality mentioned above, holding a provider's certificate was positively correlated with a provider's correct diagnosis rate, whereas a provider's income was negatively correlated with their correct diagnosis rate. Regarding the rate of correct treatment (Appendix Figure 2), NRQE was the only significant correlate: Providers who conducted 1 additional NRQE were 5% points more likely to offer correct treatment.

### Quality of the healthcare system in managing T2D

**Figure 5:**
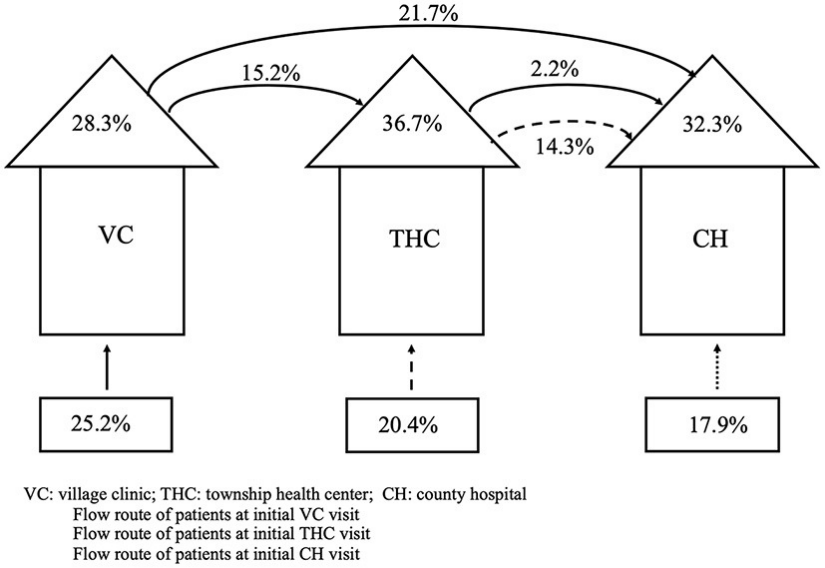
Quality of the rural healthcare system assessed through its ability to correctly treat type 2 diabetes. Source: Author's survey. VC, Village Clinic; THC, Township Health Center; CH, County Hospital.

To examine the system-level quality of T2D care, we combined provider T2D care quality, referral rates, and patient sorting behavior to generate a model that displayed the treatment of diabetes at each facility and any subsequent patient referral to higher-level facilities under the rural healthcare system (Figure 5). From our collected data on patient sorting behavior, we found that 39% of T2D patients first visited a VC, while 32% first visited a THC, and 29% went directly to a CH. Using the data from Table 2, we attributed correct treatment rates of 28.3% for VCs, 36.7% for THCs, and 32.3% for CHs. Among the patients whose symptoms were not treated at a VC, 15.2% were given a referral to a THC, and 21.7% were referred to a CH directly. Among the 15.2% of patients who transferred to a THC from a VC, 2.2% were subsequently referred to a CH. For patients who visited a THC initially, 14.3% were referred to a CH. Using this model, we were able to calculate the probability that an average rural diabetes patient will receive the correct treatment under the rural healthcare system; this probability came out at 23.7%. Detailed information on the calculation can be found in Appendix Table 2.

## Discussion

To the best of our knowledge, this is the first study to evaluate the quality of T2D care using the SP method in rural China. Overall, findings from this study indicate poor healthcare quality among rural healthcare providers in China. Regarding the quality of the diagnostic process, on average, less than a third of the recommended diagnostic examinations and questions for T2D were used during the interactions between SPs and providers. Most rural providers correctly diagnosed T2D; however, there existed large gaps in the correct diagnosis rate between providers in VCs (who correctly diagnosed 69.6% of SP cases) and providers in THCs and CHs (who gave correct diagnoses in almost 100% of cases). Additionally, despite these differences in correct diagnosis rates, the differences between the providers' treatment plans were small. Moreover, a provider's age, their income, whether they had a practicing certificate, their patient volume, and their level of training were all correlated with their diagnostic process quality, while the diagnostic process quality was the main significant correlate for the quality of diagnosis and treatment of T2D. Finally, when we assessed the overall quality of the rural healthcare system, we identified that the quality of correct treatment prescribed for a T2D patient was <30%.

Due to different quality assessment methods and quality indicators used, our results cannot be directly compared with the results of other studies. For example, one study from rural Uganda used both medical records and patient interview methods to evaluate the quality of the healthcare system, the process of case management (medical examinations in medical records), and patients' health outcomes (13). Another study from Italy employed a continuous *Q*-score to evaluate the quality of healthcare (23). Although we cannot compare our results directly to these studies, we note that two studies from China and Switzerland, using different indicators for quality of care, found overall low quality in the case management process of T2D, which is consistent with our results (24, 25). In addition, using SPs in this study allowed us to record all patient-provider interactions as well as to observe real diagnostic questions asked by providers. Our study found that two essential questions for diagnosing diabetes, specifically questions regarding weight changes and if there was a family history of diabetes, were not frequently asked by providers during the SP interactions, especially by providers from VCs and THCs. We believe that this finding may be helpful when designing interventions for improving the process quality of diagnosing and treating T2D for providers in China's rural healthcare system.

Surprisingly, we found that both process and treatment quality had no statistical difference among the three levels of providers, which was not consistent with previous research. The reason for this may be 2-fold. First, the national policy of “strengthening primary healthcare” with core responsibilities in preventing and managing chronic diseases might have improved the quality of primary healthcare for providers from VCs and THCs (26). Second, we used the SP method to assess the provider's actual clinical behaviors of T2D care instead of assessing their clinical knowledge, which was the measure most frequently used by previous research (13, 27). Additionally, although a knowledge gap may exist between providers from different tiers, clinical behaviors may not change when clinical knowledge increases (28). Therefore, the knowledge-to-real practice gap may be an important reason behind the non-significant difference observed among the three levels of providers, and should be taken into consideration in further quality improvement research.

Our results also showed that providers who had earned a practicing certificate were the ones most strongly correlated with providing better diagnosis process quality, which is consistent with results from previous diabetes healthcare research (29) and from healthcare quality evaluations done in rural China (19). However, some discrepancies with the existing research exist. In one notable example, contrary to findings from Hong Kong primary care settings, where higher patient volume was found to hamper the quality of diabetes care (30), our study's results indicated that higher patient volume was positively correlated with diagnosis process quality. This finding is consistent with evidence from a worldwide systematic review and meta-analysis (31). We believe that a potential reason for this discrepancy may be due to the fact that in Hong Kong, clinics with lower patient volume often have better continuity of care, and thus offer better care for patients with diabetes (30, 32). However, in our study, patient volume was evaluated on the provider level, not on the institutional level. Additionally, prior research has indicated that local providers are more likely to be visited by rural residents in China for primary care than doctors in large hospitals (33). Due to this, we believe that providers with higher volumes of diabetes patients (resulting from higher patient volume in general) are more likely to diagnose and treat diabetes effectively, as they are more likely to develop experience-based expertise for the diseases they frequently see and treat (34). This finding thus leads us to make the suggestion that quality-control measures, such as disease-specific clinical training, should be provided to and emphasized for rural clinicians experiencing less patient volume.

Regarding negatively-correlated factors, a provider's age was negatively associated with the NRQE used during interactions, which is in agreement with results from past studies (35). We also found that providers who participated in online training were less likely to conduct more NRQE. This is an interesting finding as, in the era of the COVID-19 pandemic, online training has become more widely accepted (36). Despite the growing acceptance of online training, however, our study provides support that more conscious efforts should be made for improving online training programs given to healthcare providers in rural China. We also believe that more research is needed to examine the effects of online training on T2D care quality.

In addition to analyzing the quality of T2D care on an individual healthcare provider level, we also evaluated the quality of T2D treatment among the whole rural healthcare system. Only 38.9% of T2D patients were treated correctly through the rural healthcare system, which is lower than the rate found in previous research assessing rural China's healthcare system's ability to manage tuberculosis (20). Additionally, T2D treatment quality across all tiers was generally low. Even treatment at the CH level, which has always been considered the highest tier of the rural healthcare system, was of lower quality than the treatment provided at THCs, the second-tier healthcare facility that connects VCs and CHs. In light of this finding, we believe that although the policy of “strengthening primary healthcare” reinforced by the Healthy China 2030 Plan announced in 2016 might have improved the quality of primary healthcare in rural areas to a certain extent (37), considering the random patient sorting behavior observed in this study, health policy should not only focus on the quality of primary healthcare, but also focus on an integrated, cooperative primary healthcare system, which fully supports health providers to improve performance (38). A team-based clinical practice intervention (i.e., integrating CHs, THCs, and VCs) and more T2D-specific treatment training for the three tiers of providers may be better ways for improving quality of T2D care and managing T2D in China's rural areas. Finally, our system-level evaluation presented a novel perspective of T2D treatment quality, which we believe could provide ex-ante estimates of the healthcare system for other non-communicable or communicable diseases.

## Limitations

Because our research is a part of the project of “Evaluating the Quality of Primary Care for the Early Diagnosis and Treatment of Non-Communicable Diseases in China's Rural Health System,” we did not employ a specific sample size calculation. Due to this study's sample size, even though we could identify the quality of care from rural providers and the rural healthcare system in China overall, we were limited to exploring a narrow range of potential contributing factors. Additionally, because the standardized script used was developed for and used by SP volunteers to present the same background and symptoms to providers, this study was unable to measure real patient characteristics. Finally, during SP interactions, all providers' clinical behaviors were assessed in a condition with unknown patients. However, we admit that this method may be limited in an acquaintance society where providers, such as village providers, live in the same rural community as patients and are thus very familiar with their patients.

## Conclusion

The quality of T2D care is poor in the rural areas of western China, especially when it came to the quality of the diagnostic process and treatment of T2D during a patient's first visit. Online training may not help to improve clinicians' quality of care for T2D. Moreover, a provider with lower patient volume may indicate that the provider will provide lower-quality care. Further research is needed to explore the causal relation. In addition, our research identified low quality of T2D care observed across the entire rural healthcare system. Finally, our research provided evidence that the rural healthcare system in western China is unable to effectively manage T2D, which may exacerbate the disease burden of T2D and lead to the development of more serious health problems when T2D is improperly treated. Further research is needed to explore potential interventions aiming at improving the T2D quality of the rural healthcare system in China and in other low-income countries.

## Data availability statement

The raw data supporting the conclusions of this article will be made available by the authors, without undue reservation.

## Ethics statement

The studies involving human participants were reviewed and approved by Medical Research Ethics Committee of Sichuan University, China (Registration No. K2019021). The patients/participants provided their written informed consent to participate in this study.

## Author contributions

HZ, SS, and SR: conceptualization. YW and RY: formal analysis. HZ and SS: funding acquisition. YW, HZ, CS, SM, ZC, LL, and SS: methodology. YW, HZ, and SR: writing—original draft. YW, RY, CS, SM, ZC, LL, SS, LP, HZ, and SR: writing—review and editing. All authors contributed to the development of the manuscript.
