# Supplementary material for: Using standardized patients to assess the quality of type 2 diabetes care among primary care providers and the health system: Evidence from rural areas of western China
Source: Front Public Health. 2022 Dec 22;10:1081239. doi: 10.3389/fpubh.2022.1081239 (PMC9815030; doi:10.3389/fpubh.2022.1081239)
Supplement: Supplementary file 1 [file Data_Sheet_1.docx]

Supplementary Material

Appendix Text 1

The consultation process was evaluated by comparing the questions and examinations that clinicians used during SP-clinician interactions to a standard checklist of China’s national clinical guidelines for type 2 diabetes case management. The national guidelines contained 17 recommended questions (including 4 essential questions), and 12 recommended checkups (including 4 essential checkups), as seen in Appendix Table 1.

**Appendix Table 1.** Recommended items and essential items for questions and examinations for diagnosing type 2 diabetes.

| **Item NO.** | **Recommended item (questions)** | **Essential item** |
| --- | --- | --- |
| 1 | Thirst | Yes |
| 2 | Weight change | Yes |
| 3 | Family history | Yes |
| 4 | Blood glucose | Yes |
| 5 | Smoking | No |
| 6 | Drinking | No |
| 7 | Numbness in hands and feet | No |
| 8 | Vision changes | No |
| 9 | Changes in drinking time | No |
| 10 | Hypoglycemia | No |
| 11 | Blood pressure | No |
| 12 | Changes in appetite | No |
| 13 | Excessive urination | No |
| 14 | Number of urinations | No |
| 15 | Drinking water | No |
| 16 | Age | No |
| 17 | Personal diabetes history | No |
| **Item NO.** | **Recommended item (examinations)** | **Essential item** |
| 1 | OGTT | Yes |
| 2 | HbA1c | Yes |
| 3 | FPG | Yes |
| 4 | Capillary blood-random blood glucose | Yes |
| 5 | BMI: height | No |
| 6 | BMI: weight | No |
| 7 | UAlb/Cr | No |
| 8 | Waist circumference | No |
| 9 | Blood pressure | No |
| 10 | Routine urine test | No |
| 11 | Capillary blood-fasting blood glucose | No |
| 12 | Capillary blood-postprandial blood glucose | No |

Consultation quality was determined by the average percentage of recommended and essential questions and checkups (ANRQC), which was calculated for each provider by the number of recommended questions and checkups they mentioned in the SP-provider interaction divided by the total number recommended by the national guidelines. Correct diagnosis was achieved if the provider diagnosed the patient with “type 2 diabetes,” “diabetes,” or “hyperglycemia.” Correct treatment was achieved by meeting either one of two conditions without prescribing harmful medications, such as glucose injections: (1) the provider gave his or her patient suggestions for diet and lifestyle changes, and recommended exercise and provided guidance for testing blood glucose levels; or (2) Condition 1 plus the prescription of appropriate oral hypoglycemic agents:

1) Biguanides-Metformin (first-line drug).

2) Sulfonylureas; ——Glyburide, Gliclazide, Glipizide, Gliquidone, Glimepiride

3) Thiazolidinediones-rosiglitazone, pioglitazone.

4) Glucosidase inhibitors-acarbose, voglibose, miglitol.

5) Non-SU insulin secretagogues-repaglinide, nateglinide.

6) Other oral hypoglycemic agents-SGLT-2 sodium glucose cotransporter inhibitor, dipeptidyl peptidase IV (DDP-4) inhibitor).

Finally, any referral to another healthcare facility and whether any follow-up appointment was requested by the provider was recorded.

**Appendix Table 2.** Calculation of the probability of correct treatment in the rural healthcare system.

|  | VC | THC | CH | Total |
| --- | --- | --- | --- | --- |
| 1. Initial visit | 25.2%*28.3% | 20.4%*36.7% | 17.9%*32.3% | 20.4% |
| 2. Referral from VC | - | 20.4%*15.2%*36.7% | 20.4%*21.7%*32.3% | 2.5% |
| 3. Referral from THC | - | - | 17.9%*15.2%*2.2%*32.3%+17.9%*14.3%*32.3% | 0.8% |
| Total | 7.1% | 8.5% | 8.1% | 23.7% |
| Notes: VC = Village Clinic; THC = Township Health Center; CH = County Hospital | | | | |

Appendix Text 2

Doctor, I have been very irritated recently, with a dry mouth and very thirsty.

Background story [Principle: If the doctor does not ask, the patient does not say anything]

Male patient: My name is Li Guoyao, 52 years old, 1.7 meters tall, and currently weight 145 kg.

1. Li Guoyao, 52 years old, born on July 13, 1967.

2. His wife Chen Xiuli, 49 years old, was born on April 18, 1970. He has been married for 28 years and has 2 children. The eldest daughter Li Ran, 25 years old. She was born in 1994 and works in Guangzhou. The youngest son Li Muzhou, 20 years old, was born in 1999 and is a university student in Chengdu.

3. There are no other diseases history. He has heavier meal tastes and loves meat and sweets. He usually smokes, half a pack a day, for 20 to 30 years, and likes to drink when he is with friends.

4. He started to feel dry mouth about 1 year ago. He always wanted to drink water and go to the toilet more often. His appetite started to increase, the frequency of eating sweets also increased, and his weight dropped by about 10 kg within a year.

5. In recent months, he has been feeling thirsty and wants to drink water. Every time he drinks a large glass of water, and he can drink 8-9 glasses a day. He feels dry and thirsty from morning to night. He urinates more than ten times a day. He usually urinates 3-4 times at night, and sometimes he sees bubbles in his urine. He eats three bowls of rice at a time, but he is hungry fast, and he feels craving for something soon after the meal, and he will eat biscuits when he is hungry. He lost 3-5 pounds in these three months.

6. He feels boring in my body in the past few months. Sometimes his eyes are a little blurry, his skin is itchy, and sometimes his hands and feet are numb, but there is no symptom of swelling. Compared with a year ago, these symptoms have become more pronounced recently.

7. Li Guoyao's mother passed away 3 years ago. There was a similar situation before, but it has not been checked. His father is alive, 76 years old this year, and has high blood pressure. No one else in the family has found a similar situation.

8. Two days ago, when he was unwell, he went to the hospital. The doctor asked him to skip a meal to take blood checkup. He didn't get the examination results because he was in a hurry to leave. He asked someone to bring it back. He took it to the doctor today.

9. Li Guoyao and his wife usually do odd jobs everywhere. They heard that there is a hospital here, so they came to see the doctor. He has medical insurance, but he did not bring the medical card today.


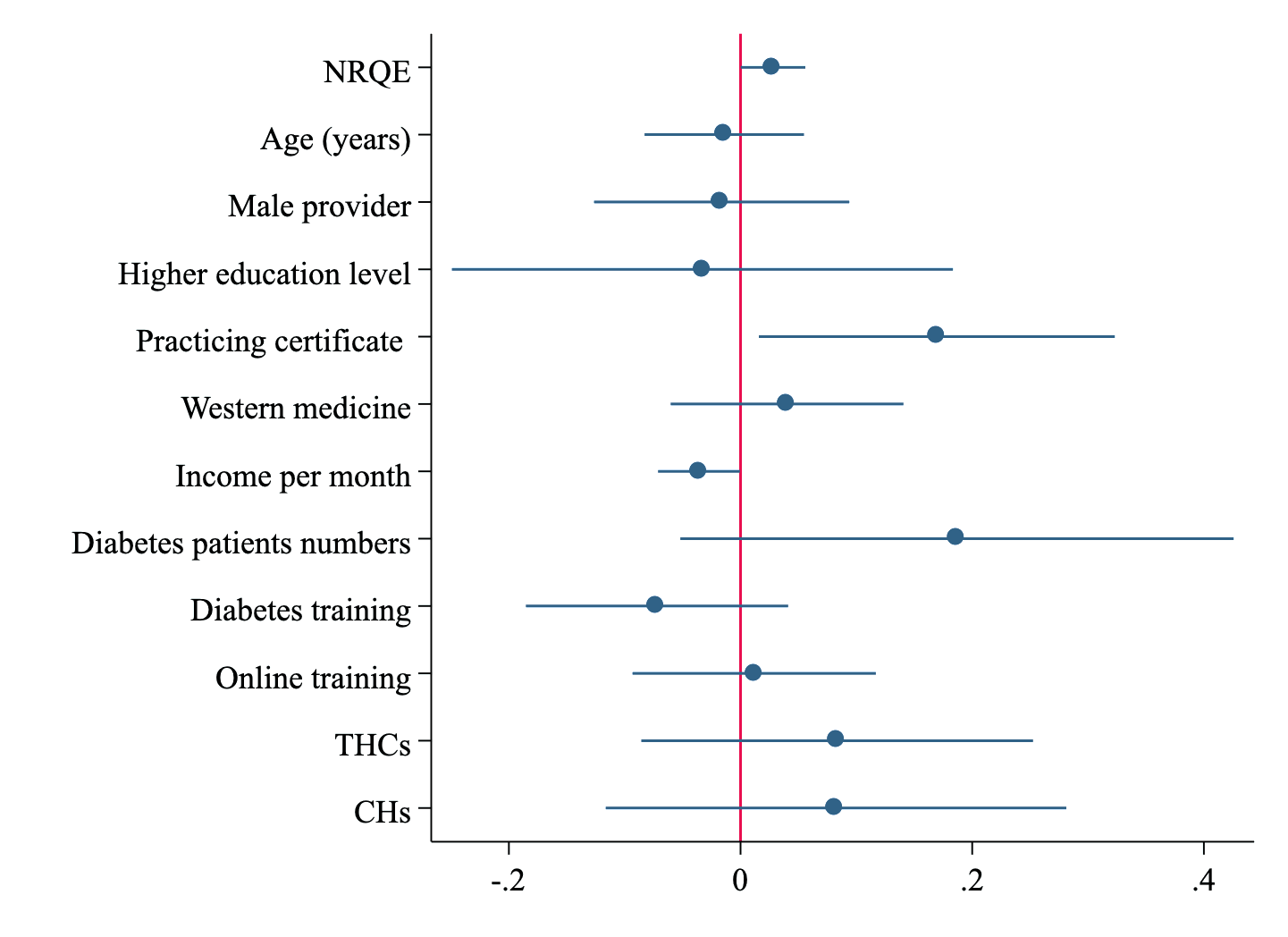


**Appendix Figure 1.** Associated factors for correct diagnosis of type 2 diabetes among rural clinicians.

*Source:* Author’s survey.

*Notes:* NRQE = number of recommended questions and examinations CH = County Hospital; THC = Township Health Center.


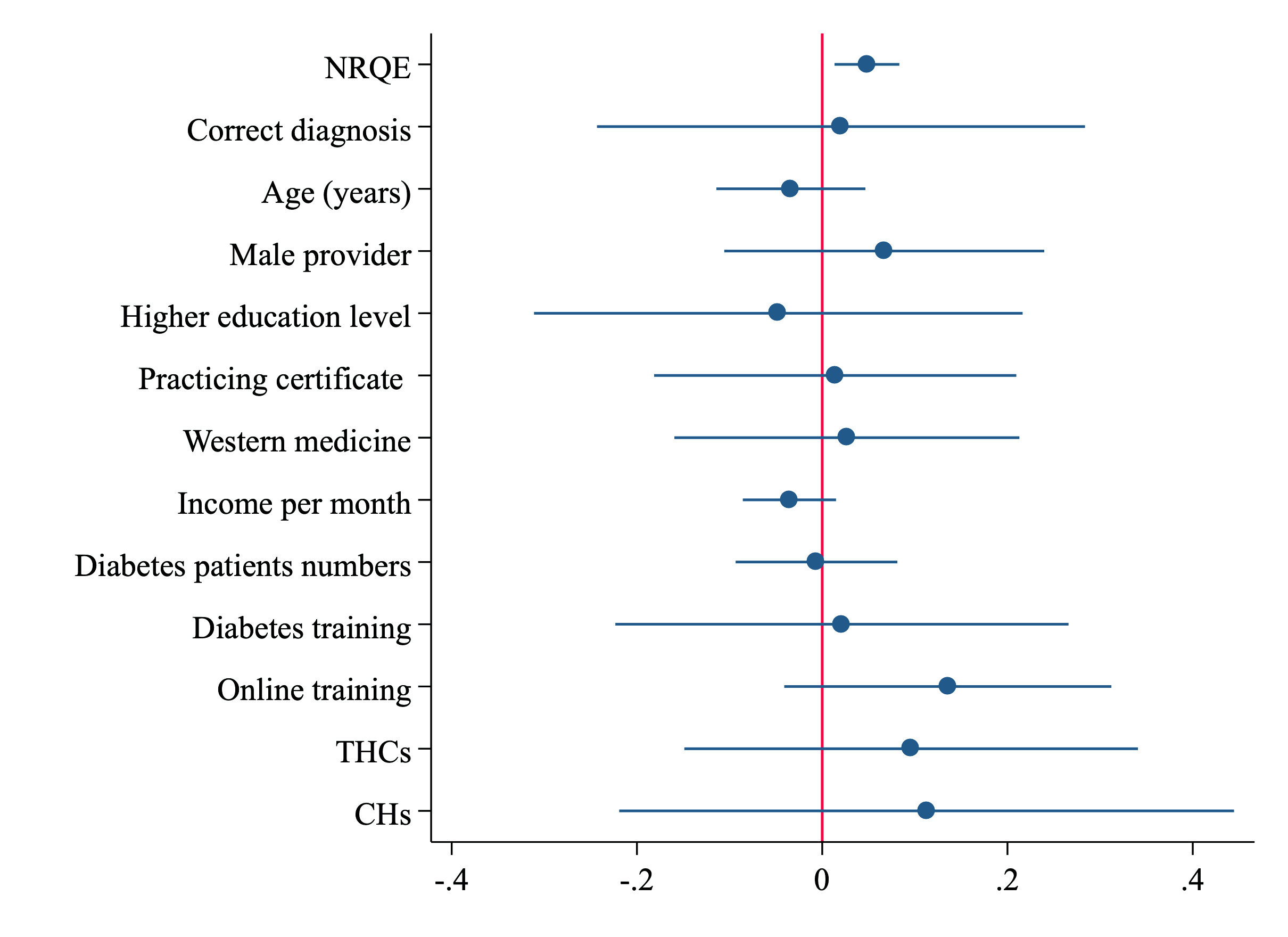


**Appendix Figure 2.** Associated factors for correct treatment of type 2 diabetes among rural clinicians.

*Source:* Author’s survey.

*Notes:* NRQE = number of recommended questions and examinations CH = County Hospital; THC = Township Health Center.
